# Apoptosis during embryonic tissue remodeling is accompanied by cell senescence

**DOI:** 10.18632/aging.100844

**Published:** 2015-11-14

**Authors:** Carlos I. Lorda-Diez, Beatriz Garcia-Riart, Juan A. Montero, Joaquín Rodriguez-León, Juan A Garcia-Porrero, Juan M. Hurle

**Affiliations:** ^1^ Departamento de Anatomía y Biología Celular and IDIVAL, Universidad de Cantabria, Santander 39011, Spain; ^2^ Departamento de Anatomía y Biología Celular, Universidad de Extremadura, Badajoz 07006, Spain

**Keywords:** programmed cell death, senescence, limb development, β-galactosidase, syndactyly, SASP, INZ

## Abstract

This study re-examined the dying process in the interdigital tissue during the formation of free digits in the developing limbs. We demonstrated that the interdigital dying process was associated with cell senescence, as deduced by induction of β-gal activity, mitotic arrest, and transcriptional up-regulation of *p21* together with many components of the senescence-associated secretory phenotype. We also found overlapping domains of expression of members of the Btg/Tob gene family of antiproliferative factors in the regressing interdigits. Notably, *Btg2* was up-regulated during interdigit remodeling in species with free digits but not in the webbed foot of the duck. We also demonstrate that oxidative stress promoted the expression of *Btg2*, and that FGF2 and IGF1 which are survival signals for embryonic limb mesenchyme inhibited *Btg2* expression. *Btg2* overexpression in vivo and in vitro induced all the observed changes during interdigit regression, including oxidative stress, arrest of cell cycle progression, transcriptional regulation of senescence markers, and caspase-mediated apoptosis. Consistent with the central role of p21 on cell senescence, the transcriptional effects induced by overexpression of Btg2 are attenuated by silencing *p21*. Our findings indicate that cell senescence and apoptosis are complementary processes in the regression of embryonic tissues and share common regulatory signals.

## INTRODUCTION

Normal development requires the coordination of growth and differentiation and the elimination of excess cells in embryonic structures. Digit formation in vertebrate embryonic limbs provides a valuable model of programmed cell death that sculpts interdigital tissue to varying degrees and confers hand/foot (autopod) morphology in accordance with the functional specialization of a species to swim (ducks, and turtles), fly (bats), or walk (chickens, humans, and lizards) [1–4]. Many studies have demonstrated that interdigit regression is a more complex process than initially thought [5–17], it includes massive apoptosis, growth arrest, and matrix remodeling of the interdigits [18]. Several recent studies have proposed that some regressive changes in the embryo include cell senescence that is similar to the senescence induced by oncogenes or senescence-inducing stimuli in adult tissues [19–21]. Digit and interdigit progenitors retain sufficient plasticity to interchange their fates until very advanced stages of development [3]. Therefore, the unraveling of the molecular machinery that determines whether a skeletal progenitor undergoes senescence and cell death or proliferates and differentiates to form a digit is of great biological relevance [22].

Factors that regulate cell cycle progression and/or tumor suppressor signals may be good candidate signals that function downstream of growth factors to control interdigital tissue regression. p53 exerts a central role in the control of most processes of cell senescence, but not in the studied examples of developmental senescence [20]. This finding suggests that other tumor suppressor genes might account for the regulation of senescence in embryonic tissues. Previous studies have demonstrated that members of the *Btg*/*Tob* family (also termed the APRO-family; [23]) are expressed in embryonic tissues ([24, 25]) and promote cell death and senescence in various cell lineages [26–28]. Deregulation of the *Btg/Tob* genes is associated with carcinogenesis and tumor growth ([27, 29–31], which is consistent with their influence on cell proliferation and cell death. Mice that are deficient in genes from this family lack a digit phenotype [32], but there is a human syndrome that is caused by the deletion of the region of chromosome 12 that contains the *Btg1* gene that results in syndactyly in toes 2-3 [33]. These findings suggest that members of this gene family may be good candidates to redundantly regulate the final stages of limb outgrowth and/or the remodeling process for digit separation.

This study examined whether apoptosis and cell senescence in embryonic systems are redundant regressive changes that are regulated by tumor suppressor genes, similar to adult tissues during ageing and cancer.

## RESULTS

### Interdigital tissue regression and cell senescence

Several programmed cell death processes in the embryo are accompanied by cell senescence [19–21], which is characterized by the irreversible loss of replicating ability. The arrest of proliferation in senescent cells is often associated with the overactivation of β-galacto-sidase (“senescence-associated β-gal”), up-regulation of the tumor suppressor gene *p21* (*inhibitor of cyclin-dependent kinases*), and the production of secreted factors, which are collectively termed the senescence-associated secretory phenotype (SASP) that reinforce and propagate senescence in autocrine and paracrine manners [42].

**Figure 1:**
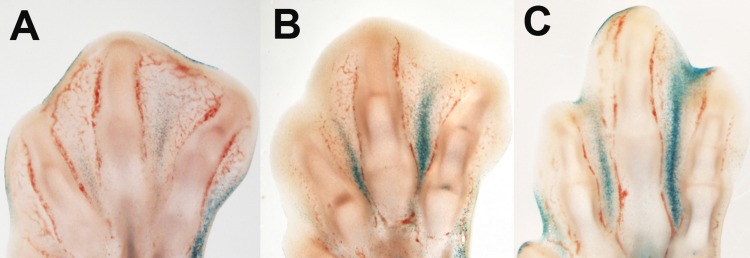
β-gal activity in the course of interdigit tissue regression in the embryonic chick. Longitudinal vibratome sections of chick limb autopods at days 7 (**A**), 7.5 (**B**), and 8 id (**C**).

The interdigital tissue expresses *p21* [43] and in the course of regression undergoes growth arrest [5, 44]. Therefore, we examined markers of cell senescence. We observed that most recognized markers of senescence were activated in regressing interdigits. Figure 1 A-C shows that β-gal activity at pH6 was very high, and it constituted a precise marker of the regressing process. *p21* was expressed at moderate levels in the interdigital mesoderm but it was up-regulated during regression (Table 1). At difference of *p21*, its usual regulator in other senescence processes, *p53* was down-regulated in the course of regression. However, *p63* and *p73*, which are the other two members of the *p53* family of tumor suppressor genes implicated in the control of mesodermal limb apoptosis [15], are also up-regulated in the course of interdigit remodeling. In addition, many characteristic members of SASP appeared intensely up-regulated. These factors included the following: cytokines, such as Interleukin 8 L1 (K60) and Interleukin 8 L2 (EMF-1); growth factors, such as Igf1, IgfBP5, *HGF*, *Tgfβ2* and *AREGB* (*Amphiregulin B*); metalloproteinases (MMP) such as, *MMP2* (gelatinase A), *MMP9*, and *ADAMTS9;* and members of the TNF signaling pathway, including *Fas* (CD95), *Tnfrsf21 (DR6)*, and *Tnfrsf 23* (Table 1). Notably, some of these factors, such as ADAMTS9 or *Tgfβs*, play a role in promoting the regression of the interdigit webs [45, 46].

**Table 1:** Transcriptional modifications of cell cycle regulators and SASP components during the regression of the third interdigit

		Expression Fold Changes		
Gene	GenBank	6 id.	7.5 id.	8 id.
**Cell Cycle Regulation**				
***p21***	AF513031	1.00 ± 0.05	1.30 ± 0.30*	3.01 ± 0.10***
***p53***	NM_205264	1.00 ± 0.06	0.57 ± 0.03**	0.40 ± 0.17**
***p63***	NM_204351	1.00 ± 0.03	2.42 ± 0.37**	4.73 ± 0.88***
***p73***	XM_417545	1.00 ± 0.04	1.67 ± 0.27*	2.37 ± 0.61*
**Senescence-Associated Secretory Phenotype (SASP)**				
***IL1b***	NM_204524	1.01 ± 0.06	1.70 ± 0.73	1.26 ± 0.43
***IL6***	NM_204628	1.00 ± 0.02	1.72 ± 0.70	1.39 ± 0.30
***IL8L1***	NM_205018	1.00 ± 0.04	11.10 ± 3.51*	20.59 ± 5.18*
***IL8L2***	NM_205498	1.02 ± 0.08	1.93 ± 0.22**	3.12 ± 0.46*
***CXCL12***	NM_204510	1.00 ± 0.03	0.43 ± 0.04***	0.53 ± 0.10**
***AREGB***	NM_001031537	1.05 ± 0.19	2.23 ± 0.43*	2.46 ± 0.71*
***HGF***	NM_001030370	1.01 ± 0.08	1.98 ± 0.20**	1.85 ± 0.37*
***TGF*β*2***	XM_003640970	1.03 ± 0.07	1.85 ± 0.25*	2.53 ± 0.53*
***IGF1***	NM_001004384	1.00 ± 0.01	5.72 ± 1.62*	7.86 ± 2.20*
***IGFBP5***	XM_004942886	1.00 ± 0.02	7.70 ± 1.86**	19.36 ± 4.44**
***IGFBP7***	XM_420577	1.00 ± 0.04	0.86 ± 0.11	0.66 ± 0.07
***MMP2***	NM_204420	1.01 ± 0.06	2.79 ± 0.45**	4.47 ± 0.78*
***MMP9***	NM_204667	1.00 ± 0.02	2.49 ± 0.44**	4.33 ± 0.91*
***Adamts9***	XM_414417	1.00 ± 0.04	6.70 ± 1.11**	22.66 ± 5.92*
***Adam17***	NM_001008682	1.00 ± 0.04	1.14 ± 0.13	1.26 ± 0.07
***FAS***	XM_421659	1.02 ± 0.04	1.88 ± 0.18***	1.81 ± 0.24*
***FASLG***	NM_001031559	1.01 ± 0.06	0.78 ± 0.16	1.59 ± 0.22
***TNFRSF21***	NM_001031103	1.00 ± 0.02	0.83 ± 0.12	1.62 ± 0.25*
***TNFRSF23***	NM_204386	1.03 ± 0.14	1.92 ± 0.31*	3.28 ± 0.88*

The values are compared with the basal interdigital expression prior to tissue regression (6 id).

*** p < 0,001;

** p < 0.01;

* p< 0.05.

### Expression of Btg/Tob genes in interdigital tissue

**Figure 2:**
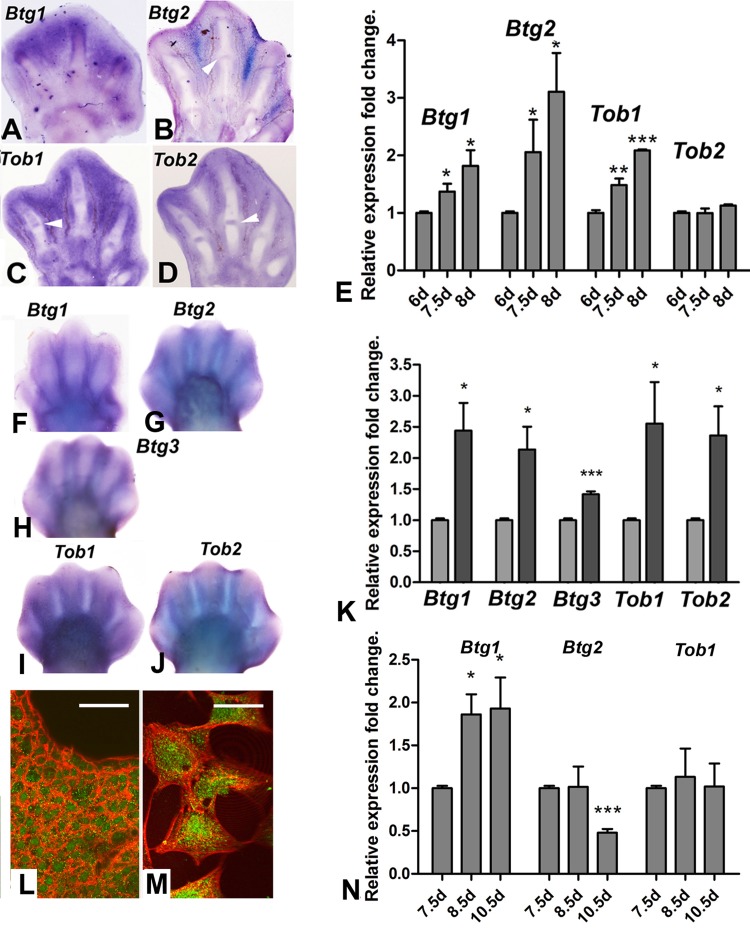
(**A-D**) in situ hybridizations showing the expression of *Btg1* (**A**), *Btg2* (**B**), *Tob1*(**C**) and *Tob2* (**D**) in the chick autopod during interdigit regression. Note that, in addition to the interdigital domains, *Tob2*, *Btg2* and *Tob1* are also expressed in the developing interphalangeal joints (arrows). (**E**) Expression level of Btg and Tob genes in interdigital tissue of chick leg during the course of remodeling. The chart shows QPCR-evaluated fold changes in the expression of *Btg1*, *Btg2*, *Tob1* and *Tob2* in the third interdigit of the chick leg bud at 7.5 and 8 id compared with their expression levels prior to the onset of tissue regression (6 id). (**F-J**) in situ hybridizations showing the expression of the *Btg/Tob* genes in the developing mouse autopod. (**K**) chart is a comparative QPCR analysis of the interdigital expression of Btg/Tob genes at day 13 p.c. (light columns) versus day 13,5 pc (dark columns). (**L-M**) immunostaining for BTG2 (green) combined with phalloidin (red) in vibratome sections of the third interdigit (**M**), and in cultured mesodermal progenitors (**M**). Note that the protein is expressed in the cytoplasm and nuclei. Scale bar in L = 100μm; Bar in M = 20μm. (**N**) shows a QPCR analysis of the expression of *Btg1*, *Btg2* and *Tob1* in the third interdigit of embryonic duck leg at equivalent stages of that of the chick in E. Unlike in the chick (compare with E), *Btg2* becomes down-regulated and Tob1 is not up-regulated over the course of tissue remodeling. ***p < 0,001; ** p < 0.01; * p< 0.05.

Members of the Btg/Tob gene family of tumor suppressor genes are expressed in early embryo, including in the limb bud [24, 25]. We investigate the potential implication of these genes in interdigit remodeling because of the role of this gene family in the regulation of proliferation of cancer cells and neural stem cells. Four members of the family, *Btg1*, *Btg2*, *Tob1* and *Tob2*, exhibited interdigital expression domains during the course of tissue remodeling in chick and mouse embryos (Fig. 2). *Btg3*, which is a member of the family that lacks known representative in avian species, is also expressed in the mouse interdigits. Figure 2 shows that not all genes exhibited identical patterns of expression in the autopodial tissues, but all were included in the zones of interdigital cell death.

Quantitative expression analysis using QPCR in interdigital tissue samples revealed that, the expression level of Btg/Tob genes, except for *Tob2*, were up-regulated as regression progressed in chick and mouse embryos (Fig. 2E and K). We compared gene expression levels in the chick and mouse legs with the embryonic duck leg, which is a model of physiological syndactyly, to select the most suitable member of the family for subsequent functional analysis in interdigit regression. *Btg1*, *Btg2* and *Tob1* were expressed in the duck leg interdigits, but *Btg2* was downregulated in the developing duck interdigits in contrast to being upregulated in chick and mouse embryos (Fig. 2N). The presence of BTG2 protein in the interdigit was also confirmed using immunolabeling in tissue sections and isolated cells (Fig. 2L and M).

We analyzed the regulation of *Btg2* in interdigit explants and mesodermal cell cultures after treatments with growth factors and signals present in autopodial tissues during interdigital tissue regression to examine the association between *Btg2* and the signaling pathways that are proposed to control interdigital cell death. Expression was down-regulated by treatments with FGF2 (0.32± 0.05 x; means ± S.D.; p<0.05) and IGF1 (0.48± 0.12 x; means ± S.D.; p<0.05) which are both survival signals for the interdigital mesoderm. Conversely, expression was up-regulated after treatments with IGFBP5 which binds and neutralizes IGF1 (2.13± 0.20 x; means ± S.D.; p<0.05) and with the IGF1 antagonist AG1024 (2.0 ± 0.09x ; means ± S.D.; p<0.05). *Btg2* expression was also up-regulated by addition of H_2_O_2_ in the culture medium to increase oxidative stress (1.49± 0.04x; means ± S.D.; p<0.01).

### Btg2 overexpression in limb mesodermal progenitors increases oxidative stress and induces cell death and cell senescence

**Figure 3:**
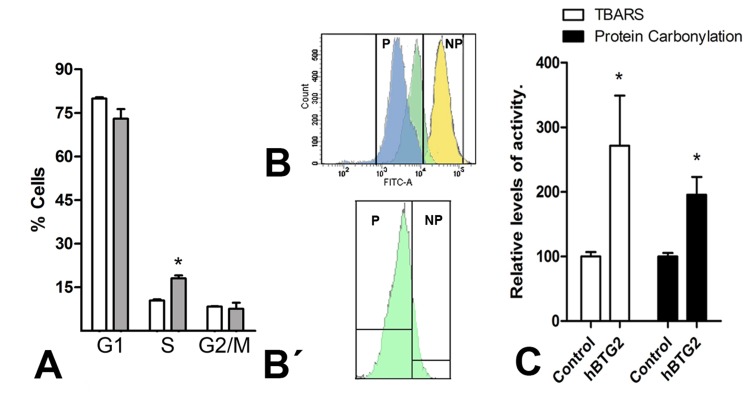
(**A**) Graphic illustrations comparing the proportion of cells at different cell cycle stages in control (white columns) versus *Btg2*-overexpressing mesodermal progenitors (grey columns) after 48 hr of culture as evaluated using flow cytometry after PI staining. (**B-B′**) Illustrates the dilution of CFSE labeling after 48 hr of culture in a representative sample of three distinct experiments. Blue: control cells; Green: *Btg2* overexpressing; Yellow: control cells maintained at 4°C. NP (non-proliferating) marks the area of the plot of no proliferation, deduced from the absence of CFSE dilution in control cells maintained at 4°C. P (proliferating) marks the area of the cytometry plot of cells in proliferation. The lower dilution of CFSE in the proliferating cell population (P) of *Btg2*-overexpressing cells (green) in comparison with control cells (blue) indicates a reduced proliferation rate. B′: detailed view of the *Btg2*-overexpressing cells in B, isolated from the other values to appreciate that a significant portion (15%) of experimental cells are non-proliferating. (**C**) Graphic representation of the levels of lipid oxidation and carbonylated proteins in control and *Btg2*-overexpressing limb mesodermal progenitors. * p< 0.05.

The extent of cell death and cell cycle profiles were analyzed using flow cytometry after propidium iodide staining in mesodermal progenitors overexpressing *Btg2* (Fig. 3). Senescence was also evaluated using flow cytometry measurement of CFSE dye dilution [40] (Fig. 3 B and B′).

Cells overexpressing *Btg2* exhibited a twofold increase in cell death compared to control cells (212.70 ± 36.70 x; means ± S.D. considering the number of dead cells in the control cultures as 100%; p<0.05) and S-phase arrest of cell cycle progression (Fig. 3A). CSFE dye dilution experiments revealed that 15% of mesodermal progenitors that overexpressed *Btg2* did not divide during our 48-hr time period in this study (15,20 ± 0.05, vs 0.20 ± 0.01 in control cultures; means ± S.D.; p<0.01; Fig. 3 B-B′).

QPCR analysis confirmed the changes induced by Btg2 at transcriptional level in limb mesodermal progenitors (Table 2). *Bcl2*, which protects from mitochondrial permeabilization (antiapoptotic), was down-regulated, and *Bak*, which induces mitochondrial permeability (proapoptotic), was up-regulated. Members of the MAF gene family of transcription factors were recently implicated in interdigital cell death [15] and these genes were also up-regulated by *Btg2*. The expression of the cell cycle regulator *p21* was up-regulated more than threefold and most components of the interdigit secretome, including: *Interleukin 8 L1*, *Interleukin 8 L2*, *HGF*, *Igf1*, *Tgfβ2*, *AREGB*, *MMP2*, *MMP9*, *Tnfrsf21*, and *Fas*, were also up-regulated (Table 2). Considering the central function of *p21* in cell senescence we analyzed whether silencing of this factor modulates the pro-senescence influence of *Btg2* overexpression. As shown in Table 2 up-regulation of most SASP components induced by Btg2 were attenuated or abrogated when the cells were transfected with h*Btg2* in combination with sh-p21.

**Table 2:** Transcriptional gene regulation in skeletal progenitors overexpressing Btg2 gene (hBtg2) and Btg2 in combination with sh-p21 (hBtg2 + sh-p21) compared with control cells transfected with empty vectors

Gene	GenBank	hBtg2(Fold changes vs Control)	hBtg2 + sh-p21(Fold changes vs Control)
**Cell Cycle Regulation**			
***p21***	AF513031	3.24 ± 0.63**	**0.54 ± 0.03** *** ##
***p53***	NM_205264	1.06 ± 0.11	0.68 ± 0.04
***p63***	NM_204351	0.83 ± 0.05	0.74 ± 0.07
***p73***	XM_417545	0.94 ± 0.10	1.19 ± 0.14
***Bcl2***	NM_205339	0.58 ± 0.03***	0.75 ± 0.04 ##
***Bak***	NM_001030920	3.20 ± 0.97*	3.81 ± 0.16*
***MAFA***	NM_205025	2.57 ± 0.17***	**1.48 ± 0.14** * ###
***MAFB***	NM_001030852	1.64 ± 0.16**	1.18 ± 0.10 #
***IL8L1***	NM_205018	6.36 ± 1.00***	**3.24 ± 0.35** ** #
***IL8L2***	NM_205498	6.07 ± 1.58**	6.42 ± 1.03**
***AREGB***	NM_001031537	3.01 ± 0.44***	1.76 ± 0.29* #
***HGF***	NM_001030370	5.46 ± 0.56***	**3.00 ± 0.16** ** ##
***TGF*β*2***	XM_003640970	2.00 ± 0.35*	0.91 ± 0.05 #
***IGF1***	NM_001004384	4.16 ± 0.58***	2.16 ± 0.14** #
***IGFBP5***	XM_004942886	0.75 ± 0.07	0.62 ± 0.03
***MMP2***	NM_204420	1.52 ± 0.15*	1.56 ± 0.04**
***MMP9***	NM_204667	1.46 ± 0.13*	1.29 ± 0.13
***Adamts9***	XM_414417	1.05 ± 0.08	**1.46 ± 0.11** * ##
***FAS***	XM_421659	2.39 ± 0.42**	**1.53 ± 0.12** ** #
***FASLG***	NM_001031559	1.11 ± 0.14	0.98 ± 0.13
***TNFRSF21***	NM_001031103	1.95 ± 0.29**	0.94 ± 0.10 ##
***TNFRSF23***	NM_204386	1.02 ± 0.11	0.77 ± 0.11

*** p < 0,001;

** p < 0.01;

* p< 0.05 statistical significance vs control cells;

### p< 0,001;

## p < 0.01;

# p< 0.05 statistical significance vs hBtg2.

Btg2 increases oxidative stress in HeLa cells [47]. Therefore, we explored whether overexpression of *Btg2* in limb mesodermal progenitors was accompanied by changes in oxidative stress. We detected a threefold increase in lipid oxidation and a twofold increase in carbonylated proteins in 2-day cultures of limb mesodermal progenitors over-expressing *Btg2* (Fig. 3C).

### Overexpression of Btg2 inhibits limb outgowth in vivo

**Figure 4:**
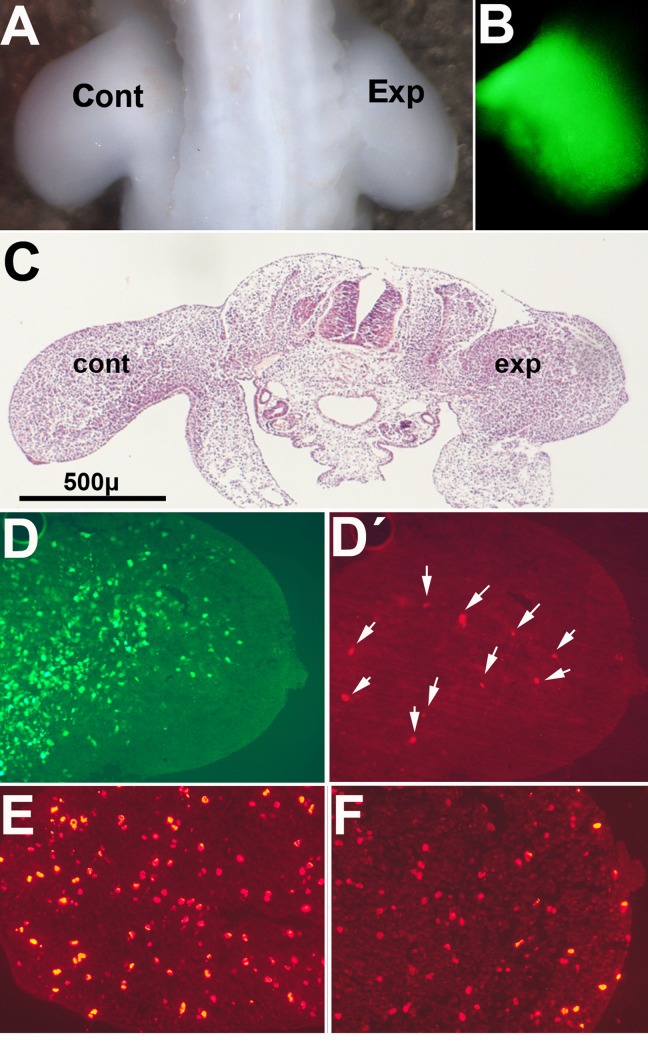
Pictures illustrate chick embryos 48 hr after electroporation of *GFP /Btg2* constructs in the right wing bud at 2 id (**A**) Dorsal view of a chick embryo showing the reduced size of the experimental limb (Exp) compared with the contralateral control (Cont). (**B**) Mesodermal expression of GFP in the limb 48 hr after electroporation. (**C**) HE stained transverse section of an experimental embryo to show the different size of the experimental limb (exp) compared with the contralateral limb (cont). (**D**) and (**D′**), show the expression of GFP (**D**) and the presence of TUNEL-positive apoptotic cells (arrows in (**D′**) in a correlative section of the experimental limb in (**C**). **E-F**, are correlative sections of the embryo in (**C**) after immunolabeling with anti-p-H3. Note the reduced number of positive cells in the experimental limb (**F**) compared with the control limb (**E**).

**Figure 5:**
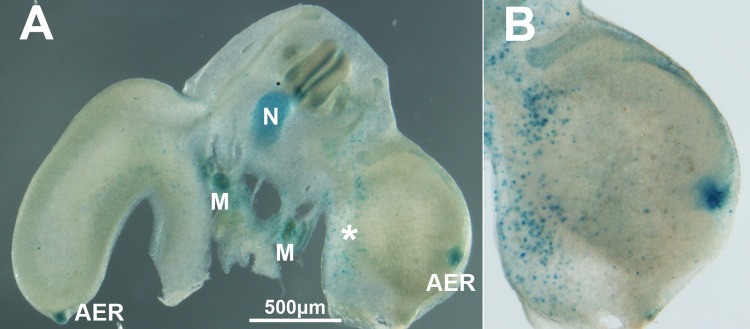
(**A**) Transverse vibratome section of an experimental embryo 48 hr after electroporation of *Btg2*. Note the abundance of cells positive for β-gal activity in the electroporated limb (*). (**B**) is a detailed view of the limb electroporated with *Btg2*. As previously reported, β-gal activity is intense in the AER of both control and experimental limbs, in the mesonephros (M) and in the notochord (N).

We overexpressed *Btg2* in the mesenchymal limb progenitors of day 2 embryos to investigate whether the antiproliferative and proapoptotic effects of *Btg2* were also induced in vivo. Electroporation at this stage is very efficient and devoid of undesired side effects [37]. This stage precedes the initiation of interdigital cell death by 4 days, but it is well known that the response of the limb mesoderm to death-inducing and survival signals is maintained during the entire period of morphogenesis [48]. Control of transfection using a GFP construct confirmed the mesodermal distribution of the electroporated genes (Fig 4B, D). Figures 4A and C show that the size of the transfected limbs was reduced compared to the contralateral control limbs. Histological analysis demonstrated the presence of a moderate number of TUNEL positive cells in the experimental limb that were not observed in the control contralateral limb (Fig. 4 D′). The number of anti-p-H3 positive mesodermal dividing cells in experimental limbs decreased 33% compared to control limbs (67,4 ± 3,6; mean values ± S.D. per field considering the number of p-H3 positive cells in the control limb as 100%; Fig. 4 E-F). Cells positive for β-gal labeling also appeared increased in experimental limbs in relation with their contralateral control (Fig. 5). As observed in the in vitro experiments these changes were accompanied by up-regulation of *p21* (1,47 ± 0.19 x; mean± S.D.; p<0.05)

## DISCUSSION

Previous studies support the coexistence of different cell death effectors that are able to replace one another during the elimination of the interdigital mesoderm [13, 49, 50]. These death effectors include caspase-driven apoptosis [12], lysosomal-driven cell death [13], and oxidative stress-driven cell death [8, 16, 51]. Our findings add changes in the characteristics of senescence in the regressing interdigits to these factors. We detected an intense up-regulation of *p21*, which is a characteristic feature of developmental senescence [21], that was accompanied by an up-regulation of characteristic components of the senescence-associated secretory phenotype (SASP) including *Interleukin 8 L1*, *Interleukin 8 L2*, *Igf1*, *IgfBP5*, *HGF*, *Tgfβ2*, AREGB (*Amphiregulin* B), the metalloproteinases *MMP2*, *MMP9* and *ADAMTS9*; and members of the TNF signaling superfamily (*Fas*, *Tnfrsf21*, and *Tnfrsf 23*). SASP components in adult and tumoral tissues function as a hierarchical network that establishes functional links between regressive changes. SASP functions include the induction of low-level inflammation accompanied by the activation of matrix proteases, the disruption of stem cell function, and the liberation of oxidizing molecules [52–54]. These processes, together with programmed apoptosis, are central events during physiological interdigit tissue regression.

We also detected an intense activation of β-gal in the regressing interdigits. This feature is a hallmark of cell senescence [21, 34]. Notably, the labeling pattern of β-gal activity in the interdigits closely correlated with progression of cell death during interdigit remodeling.

**Figure 6:**
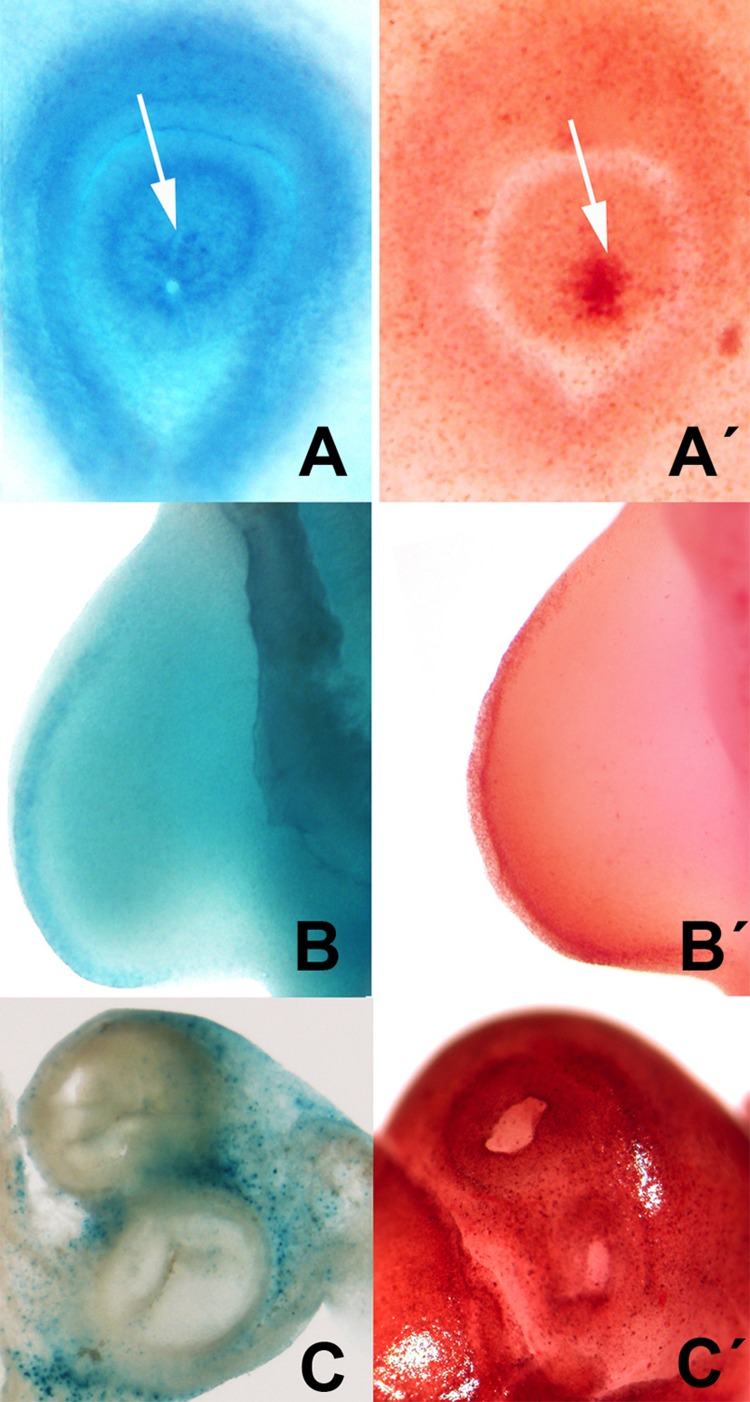
Selection of areas of embryonic programmed cell death showing the parallelism between the distribution of neutral red vital staining (**A′, B′, C′**) and β-gal activity (**A, B C**). **A-A′**: cell death (arrows) during the closure of the lens in chick embryos at 2.5 id. **B-B′** cell death in the AER in the embryonic limb at id 3.5. **C-C′**, cell death in the root of the main arteries of the embryonic heart at id 7.5.

This correlation was also observed in other areas of embryonic cell death including the closure of the lens, limb AER, and the developing heart (Figures 5 and 6).

Our finding also revealed that members of the *Btg*/*Tob* gene family of antiproliferative, tumor suppressor factors [55] were expressed in the interdigital tissue, which suggests a redundant participation in the control of tissue remodeling. The *Tob* and *Btg* genes have evolutionarily conserved domains in their amino terminal regions that contribute to their antiproliferative activities [23, 56, 57]. Furthermore, each family member participates in distinct physiological processes via a variety of molecular mechanisms, including regulation of DNA-binding of sequence-specific transcription factors, interaction with intracellular regulatory factors, and modulation of mRNA turnover [31]. *Btg2*, which is the founding member of the Btg/Tob gene family, replicated the molecular and cellular changes in limb mesoderm during interdigital tissue remodeling, as observed in tumoral tissues [26, 28]. These changes were induced by gain-of-function experiments in vivo and in vitro and included apoptosis, transcriptional modification of cell senescence characteristics, arrest of cell cycle progression, and oxidative stress. The proapoptotic influence was associated at the transcriptional level with the down-regulation of *Bcl-2* and the up-regulation of *Bak*. Therefore, the expression ratio between *Bcl2* and *Bak*, which determines cell fate (apoptosis vs survival, [58]), was shifted toward apoptosis. Cell cycle arrest is accompanied by intense up-regulation of *p21*, as observed in other senescence processes [19, 21]. Notably, 15% of cells over-expressing *Btg2* did not replicate during the 48 hr period that was analyzed in this study, which supports the induction of cell senescence. *Btg*2 overexpression also induced the expression of the SASP components in the interdigit, including *Interleukin 8L1, Interleukin 8L2*, *Igf1*, *HGF*, *Tgfβ2*, *AREGB*, *MMP2, MMP9*, *Tnfrsf21,* and *FAS*. Our findings show that p21 exerts a central role in these effects. Hence, up-regulation of many components of the SASP transcriptome by overexpression of *Btg2* is abrogated or reduced when *p21* is silenced by co-transfection with a sh-RNAi against p21.

Oxidative stress plays a central role in interdigit regression [8, 16, 51]. Our study indicate that overexpression of *Btg2* doubled the level of protein carbonylation and tripled the level of lipid peroxidation. We also demonstrated that *Btg2* was up-regulated when oxidative stress increased in limb mesoderm in cultures containing H_2_O_2_. This finding suggests that the local increase in ROS observed in the regressing interdigit [51] establishes a positive feedback loop that promotes and/or maintains the expression of this antiproliferative factor.

One remarkable feature of the regulation of interdigital cell death is the combined participation of various death mechanisms. The coordination of these dying routes is not known. This study provides a new mechanism that promotes cell senescence and apoptosis. We demonstrate that *Btg2* was down-regulated in cultures treated with FGF2 or IGF1, which are major antiapoptotic factors implicated in interdigital cell death [59–63] and up-regulated after chemically blockade of IGF signaling and when mesodermal progenitors were cultured in the presence of IGF binding protein-5. The interdigital expression of various IGF binding proteins that antagonize IGF1 [59] and the decay of FGF production by the AER at the end of limb morphogenesis together with the local increase in ROS may all contribute to the physiological up-regulation of *Btg2* in the regressing interdigit.

In summary, this study highlights the contribution of a senescence-like process during the regression of interdigital tissue and delineates the involvement of *Btg2* in cooperation with *p21*, in the promotion of cell cycle arrest, caspase-dependent cell death, and transcriptional up-regulation of components of the senescence-associated secretory phenotype. Our unpublished observations also indicate that other members of this family, such as Btg1 and Tob1 also promote cell death in the limb mesoderm, which explains the absence of an overt digit phenotype in mice that are deficient for members of this gene family.

## METHODS

### Animal models

We used Rhode Island chicken embryos from 2 to 8.5 days of incubation (id), Royal Pekin duck embryos from 7.5 to 11 id, and C57BL6 mouse embryos from 12 to 14 days post-coitum (p.c.).

### In situ hybridization

PFA-fixed specimens were treated with 10 μg/ml of proteinase K for 20-30 minutes at 20°C. Hybridization with digoxigenin-labeled antisense RNA probes was performed at 68°C. An alkaline phosphatase-conjugated anti-digoxigenin antibody (dilution 1:2000) was used (Roche). Reactions were developed with a BCIP/NBT substrate (Roche).

The probes for chick *Btg1, Btg2, Btg4, Tob1 and Tob2*; duck *Btg1*, *Btg2*, and *Tob1*; and mouse *Btg1*, *Btg2*, *Btg3*, *Btg4*, *Tob1* and *Tob2* were obtained from PCR.

### Mesodermal cultures

Mesodermal progenitors were obtained from the progress zone region of chick leg buds at 4.5 id (25 HH) or from the interdigital regions at 5.5 id. Cells were dissociated and suspended in DMEM with 10% fetal bovine serum, 100 units/ml penicillin and 100 μg/ml streptomycin. Cell death, proliferation and transcriptional changes were analyzed in subconfluent monolayer cultures of control and *Btg*2-overexpressing mesodermal cells.

Regulation of *Btg2* was investigated in high density cultures after the following treatments: FGF2 (66 ng/ml.; Peprotech); IGF1 (100 ng/ml.; GenScript); IGFBP5 (3 μg/ml.; Origene); the inhibitor of IGF signaling AG1024 (15 μM; sc-205907, Santa Cruz Biotechnology); and H_2_O_2_ (0.7 mM.)

### β-gal activity and immunolabeling

The β-galactosidase activity assay [34] was performed at pH 6 in vibratome sections of limb autopods fixed in 4% glutaraldehyde.

Immunolabeling for BTG (anti-Btg2; sc-33775, Santa Cruz Biotechnology) was performed in samples fixed in 4% PFA. Detection of mitotic cells was performed using immunolabeling with an anti-phospho-histone H3 antibody (Milipore). Counterstaining was performed using rhodamine-phalloidin (Sigma) or DAPI (Vector Laboratories).

### Cell nucleofection

We used constructs for the human *Btg2* gene cloned into the pCMV6-XL5 vector (SC115914, Origene) and also pCAGGS-GFP [35] for gain-of-function experiments. p21 gene silencing was performed by electroporation of a short hairpin RNAi (sh-p21), cloned into the pcU6-1-shRNAi (a generous gift of Dr Tim J Doran) as described by [36]. The efficiency of electroporations was confirmed using QPCR and immunohistochemistry.

For *in vitro* experiments, cells were electroporated using the Eppendorf Multiporator system (Eppendorf) following the manufacturer's instructions. Control nucleofections using the empty vector were performed in all experiments.

For in vivo experiments, the limb bud was electroporated at day 2 of incubation (stage 13 HH) with a mix of 0.1% Fast Green (Sigma®), 1 μg/μl pCAGGS-GFP, and 8 μg/μl of *hBtg2-* pCMV6-XL5 according to the Gros and Tabin procedure [37]. Limbs electroporated with pCAGGS-GFP alone developed normally and were used as controls. Identification of *GFP* labeling was used to assess the spatial distribution of electroporated cells in vivo.

The gross morphology of limbs overexpressing *Btg2/GFP* was examined in paraffin wax or vibratome sections. These sections were also used for TUNEL and β-galactosidase activity assays, and p-H3 immunolabeling, to detect cell death, cell senescence and cell proliferation respectively.

### Oxidative stress assays

Oxidative stress was evaluated via quantification of lipid oxidation and protein carbonylation in control and Btg2-overexpressing cultures of limb mesodermal progenitors. Cells were lysed using 20 mM potassium phosphate lysis buffer containing 0.1% Triton and 150 mM NaCl. Cell lysates were clarified of cellular debris by centrifugation (10000xg) at 4*00BA*C for 10 min, and the supernatants were used in the biochemical assays.

Lipid oxidation was evaluated using the thiobarbituric acid based assay (TBARS) according to the method described by Ohkawa et al. [38] Protein carbonylation was determined using a 2,4-dinitrophenyl-hydrazine based assay [39]. Data were adjusted in accordance with the total amount of proteins measured in our samples. Data in experimental samples were compared to results obtained in control samples, which were considered 100%.

### Flow cytometry

Control and *Btg2-*overexpressing cell cultures were dissociated by treatment with trypsin EDTA (Lonza). One million cells were used in each test. For propidium iodide (PI) staining the cells were washed twice in PBS and fixed in 90% ethanol. Samples were incubated overnight at 4°C with 0.1% sodium citrate, 0.01% Triton X-100 and 0.1 mg/ml PI. The cell suspension was subjected to flow cytometry analysis in a Becton Dickinson FacsCanto cytometer and analyzed using Cell Quest software.

The pattern of cell division was evaluated in control and experimental cells by measuring CFSE dye (Molecular Probes) dilution after two days of proliferation [40]. Prior to culturing the isolated cells were incubated in10 mM CFSE in DMSO diluted at 1/10,000 in PBS for 10 min. The decay in labeling intensity relative to cell proliferation was analyzed using flow cytometry. The basal labeling level was estimated from non-proliferating cells maintained at 4°C.

### Real time quantitative PCR (Q-PCR) for gene expression analysis

Total RNA was extracted using the NucleoSpin RNA kit (Macherey-Nagel). First-strand cDNA was synthesized using random hexamers and the High Capacity cDNA Reverse Transcription Kit (Life Technologies). The cDNA concentration was adjusted to 0.5 μg/μl. SYBRGreen (Life Technologies)-based QPCR was performed using the Mx3005P system (Stratagene). Rpl13 was chosen as the normalizer in interdigital samples and *Gapdh* in cultures. Mean values for fold changes were calculated. Expression level was evaluated relative to a calibrator according to the 2^−(ΔΔCt)^ equation [41]. Each value represents the mean ± SEM of at least three independent samples obtained under the same conditions. Data were analyzed using Student's *t* test or ANOVA followed by Bonferroni test for post-hoc comparisons. Statistical significance was set at p < 0.05. Q-PCR specific primers for chick, duck and mouse genes analyzed in this study would be provided upon request.
